# Testosterone Increases: Sodium Reabsorption, Blood Pressure, and Renal Pathology in Female Spontaneously Hypertensive Rats on a High Sodium Diet

**DOI:** 10.1155/2011/817835

**Published:** 2011-04-26

**Authors:** Bei Liu, Daniel Ely

**Affiliations:** Department of Biology, The University of Akron, Akron, OH 44325-3908, USA

## Abstract

Estrogen (E) and testosterone (T) are important in the sexually dimorphic pattern of blood pressure (BP) development. The goal was to examine the effects of endogenous E and exogenous T in the development of hypertension in female spontaneously hypertensive rats (SHR) on a high sodium diet. Female SHR (*N* = 27, 5-week) were divided into four groups: (1) control (*n* = 8), (2) ovariectomized (OVX, *n* = 26), (3) testosterone implants with intact ovaries (T, *n* = 6), and (4) ovariectomized + testosterone implants (OVX+T, *n* = 7). T was given immediately after OVX and replaced every two weeks and they were fed a 3% NaCl diet. BP was measured weekly and plasma norepinephrine (NE) analyzed by HPLC. OVX+T females exhibited the greatest elevation in BP (190 ± 4.0 mmHg) compared to controls at 15 weeks of age (140 ± 3.4 mmHg, *P* < .001) and a pattern of hypertension development similar to that of male SHR. Females with T treatment showed evidence of glomerulosclerosis. In conclusion, T accelerated the development of hypertension similar to the BP pattern observed in males; the presence of ovaries attenuated the T induced increase in BP; T increased renal sodium reabsorption, and T increased glomerulosclerosis.

## 1. Introduction

A sexual dimorphism in blood pressure (BP) exists in most mammalian populations with males having higher and more rapid elevation of BP than females [1, 2]. In most strains of rats used to study, BP this sex difference exists, such as in normotensive Sprague-Dawley rats [3], in Dahl salt-susceptible and salt-resistant rats [4], and in spontaneously hypertensive rats (SHRs) [5]. Also, there are gender differences in BP development in human hypertension, with males having higher BP at an earlier age than females. However, the prevalence of hypertension is actually higher in age-adjusted females, and the current rise in hypertension prevalence is primarily driven by women [6]. The triggers for gene and physiological system activation regulating BP, like the renin angiotensin system, the sympathetic nervous system and oxidative stress pathways are most likely regulated differently in females than in males [7].

Both androgens and estrogens are being used more widely, and studies need to be done to properly assess benefits and risks on the cardiovascular and renal systems. Androgen use is occurring in women as a treatment for sexual dysfunction and bone mass loss and to enhance cognition in aging [8]. However, more studies need to explore androgen dosages in females across the life span since requirements and physiological effects change [9, 10]. One of the potential risks for long-term androgen use in women is associated with impaired vascular reactivity which was found in genetic females (transgender population) [11]. Although there have not been many studies of chronic androgen use in females, Van Kesteren et al. [12] showed that in 293 transgender cases there were no increased risks of heart disease compared to the general population, but physiological measures and renal disorders were not examined. Nonhuman primate studies show that ovariectomized females treated with androgens for 8 months had increased coronary artery plaque size compared to controls, and this effect was not explained by lipid changes [13]. To further characterize the relationship between sex steroids and BP, gonadectomy and hormone replacement experiments have been done. Castration lowers BP in males, and T replacement increases BP and renal pathology [14–16]. Not only T, but also the presence of androgen receptors influence the development of hypertension. Our laboratory showed that hybrid SHR (the male offspring of King Holtzman females carrying the testicular feminized mutation bred with an SHR male) without functional androgen receptors had significantly reduced BP compared to those with functional androgen receptors [17].

Although most of the literature supports the concept that T potentiates while E attenuates the development of hypertension, controversy exists regarding the mechanisms played in BP regulation by both steroids [18]. Also, our laboratory and others have explored the effect of varying sodium diets on the development of high BP in SHR and WKY animals [19]. Our results and others have focused primarily on the males and only a few studies have examined the effects of E and T in females on a high sodium diet. The present study tested the hypothesis that exogenous T will increase BP in SHR females with a male pattern of BP on a high sodium diet which will result in reduced sodium excretion and renal pathology.

## 2. Materials and Methods

### 2.1. Animals, Diet, and Treatments

Female SHR (*N* = 27) were obtained from Harlan Sprague Dawley (Indianapolis, Indiana) at four weeks of age. At five weeks of age, animals were housed two per cage (polyethylene animal cages, 50 × 38 × 20 cm) with steel lids and bedding changed weekly (P. J. Murphy, Forest Products, Montville, NJ). They were maintained at a constant temperature (25–27°C) and 40–50% humidity. Lighting was controlled by an automatic timer which allowed illumination from 6:00 AM to 6:00 PM daily. All rats were fed a high sodium rat chow (3% NaCl) made from fine powdered rodent blox (Laboratory Rodent Diet 48904-F4, Harlad Teklad, Madison, Wisconsin). The purpose of the high sodium diet was to compare SHR female BP and pathology responses to those of SHR males previously studied on the same high sodium diet [19]. Food and tap water were available *ad libitum*. All animals were treated humanely following NIH guidelines, and all protocols were approved by The University of Akron IACUC.

The rats were randomly divided into four groups at five weeks of age and received the following treatments: sham operation with blank implants = controls (C, *n* = 8), ovariectomized (OVX, *n* = 6), testosterone implant (T, *n* = 6), and ovariectomized and T implantation (OVX + T, *n* = 7). The implants were changed every 2 weeks under anesthesia (Brevital Sodium 50 mg/Kg, i.p., Eli Lilly and Co. Indianapolis, IN). The OVX groups were ovariectomized before they are considered adults which is about 8 weeks of age. The sham rats given T may have had their estrous cycle influenced, but smears for the estrus stage were not taken. When BP was examined across the estrous cycle, it did not change based on unpublished data from another study in our lab.

 Implants were made of silastic medical grade tubing (1.19 mm, i.d., 16 mm, length; Dow Corp. Midland, MI), packed with testosterone propionate (T, 20 mg/capsule, Sigma, St. Louis, MO) and sealed with silastic adhesive type A as previously described (Dow Corning Corp. Midland, MI) [17]. This length of implant delivered a T dose that was similar to control levels of T and used previously in BP and androgen receptor experiments [17]. Implants were incubated in an albumin buffered primer solution at 5°C for 12 hours and soaked in 75% ethanol alcohol for 2 hours before insertion. Sham implants were given to the OVX group.

### 2.2. Blood Pressure and Kidney Histology

 BP was measured weekly, from 6 to 15 weeks of age, by the tail-cuff method using a sphygmomanometer and physiograph recorder (Narco, Bio-System, Houston, TX) [20]. Rats were individually placed in a preheated chamber (39°C) for 15 minutes. Rat restrainers were used to keep animals in position while their BP was taken. The mean of five successive BP measurements was used as the average BP for each rat. Body weight was determined weekly on the same day as the BP was measured. All animals were sacrificed after 15 weeks and kidneys weighed and processed (Tissue-Tek V.I.P. 1000 processor) for histology and embedded in paraffin [16]. Sections were cut at 5 um, placed on glass slides, and stained with hematoxylin and eosin. A qualitative scale of 0, I-II, and III-IV was used to score glomeruli, with 0 being normal having a glomerulus that filled Bowman's capsule, I-II representing moderate glomerulosclerosis where the glomerulus had shrunk to 20–40%, and III-IV representing severe glomerulosclerosis where the glomerulus had shrunk to 40–60%, [16]. Scoring of 100 glomeruli per kidney was done by three individuals blinded to the treatments and the scores averaged.

### 2.3. Plasma Norepinephrine and Sodium Measures

All blood samples were collected on ice and centrifuged (5000 × G, 10 min). Plasma was obtained and stored at −70°C for further analysis. Plasma NE was measured at 15 weeks of age by HPLC with electrochemical detection as previously described [21]. Briefly, animals were taken in their home cage to a surgical suite and anesthetized with Brevital and a retro-orbital puncture performed. About 2.5 mL of blood was collected in less than a minute. The plasma level of NE is high compared to collection by other techniques perhaps because of the movement of the cages to a new room, application of an anesthetic, and the fact that animals are on a high sodium diet, which elevates plasma NE [22]. We get plasma NE values of about 1500 pg/mL from male SHR in a colony environment compared to 2500–3000 pg/mL in these experiments [21]. To collect urine for sodium excretion analysis, animals were put into metabolic cages for 24 hr at 8 and 15 weeks. Urinary sodium was measured using a sodium sensitive electrode (Fisher, Pittsburgh, PA).

### 2.4. Statistical Analysis

Differences in BP, sodium excretion, pathology scores, kidney weight, and T levels were measured at different time intervals, and the statistical comparisons were analyzed between the experimental and control groups. Two-way ANOVA with repeated measures followed by appropriate *t*-tests (Kruskal-Wallis) was used for the comparison between control and treatment groups and for the renal pathology one-way ANOVA and follow-up Dunn's method pair-wise comparisons (Sigma Stat, Jandel Scientific, Corte Madera, CA). All data were presented as mean ± SEM. The significance was set at the level of *P* < .05.

## 3. Results

**Figure 1:**
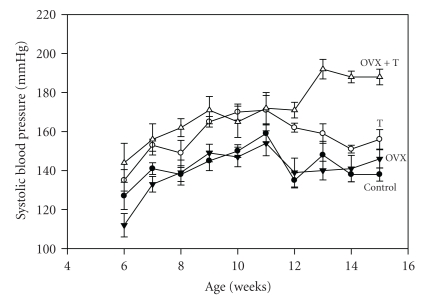
Systolic blood pressure of female SHR by treatment and time (means ± SEM). Two-way repeated measures ANOVA: treatment-*F* = 19.6, *df* = 3, *P* < .001, age-*F* = 14.7, *df* = 9, *P* < .001, interaction-*F* = 2.3, *df* = 27, *P* < .001. Solid circles: controls (*n* = 8), solid triangles: ovarectomy-OVX (*n* = 6), open circles: testosterone treated-T (*n* = 6), open triangles: ovarectomy and testosterone treatment-OVX+T (*n* = 7).

**Figure 2:**
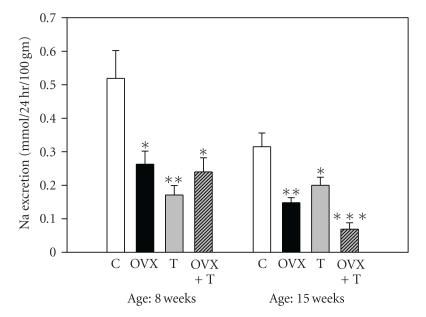
Urinary sodium excretion (24 hr) by age and treatment (means ± SEM). Two-way repeated measures ANOVA: treatment-*F* = 9.1, *df* = 3, *P* < .001, age-*F* = 3.1, *df* = 9, *P* < .05, interaction not significant. Open bars: controls (C, *n* = 8), solid black bars: ovarectomy (OVX, *n* = 6), gray bars: testosterone treated (T, *n* = 6), striped bars: ovarectomy and testosterone treatment (OVX+T, *n* = 7) (*P < .05, **P < .01, ***P < .001 compared to control).

BP change over time by group is shown in Figure 1 (two-way ANOVA: treatment: *F* = 19.6, *df* = 3, *P* < .001, age: *F* = 14.7, *df* = 9, *P* < .001, interaction: *F* = 2.3, *df* = 27, *P* < .001). BP was highest at 15 weeks of age in the OVX+T group (190 ± 4 mmHg) compared to controls (138 ± 3 mmHg, *P* < .001). The T group also had elevated BP compared to controls at 15 weeks (156 ± 5 mmHg and 138 ± 3 mmHg, resp., *P* < .05). The BP of the OVX group was not significantly different than the controls at 15 weeks (146 ± 5 mmHg compared to 138 ± 3 mmHg).  Figure 2 shows that sodium excretion in all treated groups was significantly reduced compared to controls at both 8 and 15 weeks ranging 50–84%. There was less overall sodium excretion with time, and at 15 weeks the OVX+T group had an 84% reduction. Two-way ANOVA with repeated measures showed a significant treatment effect (*F* = 9.1, *P* < .001) but not an age effect (*F* = 3.1, not significant), and there was no significant interaction. 

**Figure 3:**
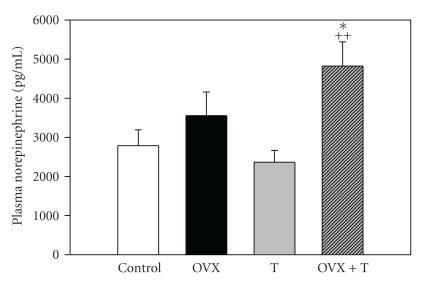
Plasma norepinephrine at the end of the experiment by treatment group (means ± S.E.M.). Open bars: controls, *n* = 8, solid black bars: ovarectomy (OVX, *n* = 6), gray bars: testosterone treated (T, *n* = 6), striped bars: ovarectomy and testosterone treatment (OVX+T, *n* = 7). One-way ANOVA: *H* = 9.6, *df* = 3, *P* < .05, *P < .05 compared to controls, ^++^ *P* < .01 compared to testosterone treatment.

With regard to plasma NE, there were both a significant treatment and age effect (treatment: *F* = 6.29, *P* < .01; age: *F* = 101, *P* < .001; no interaction significance, Figure 3). The treatment effect was consistent across time in that OVX+T had increased NE at 7 weeks (*P* < .05) and at 14 weeks (*P* < .05) compared to respective controls. At 7 weeks, there was a significant decrease in NE in the OVX group which was not observed at 14 weeks.

**Figure 4:**
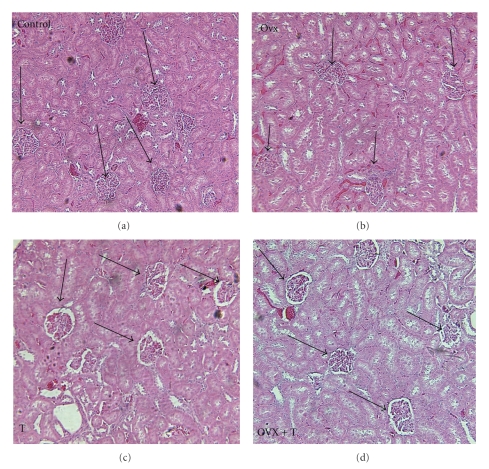
Representative renal micrographs stained with hematoxylin and eosin by treatment groups (controls, OVX: ovarectomy, T: testosterone, OVX+T: ovarectomy and testosterone) showing glomerulosclerosis (arrows point to glomeruli, magnification = 100x).

Figure 4 is a representative micrograph of a kidney from each of the four treatment groups. The glomeruli were degenerating creating a greater distance between them and Bowman's capsule with scarring of the glomerulus.

Table 1 shows the scoring of the glomeruli by treatment group. In all groups, there was some glomerular change most probably due to the high sodium diet. One-way ANOVA was significant between treatments (*H* = 63.4, *df* = 11, *P* < .001). The controls and OVX groups showed that most of the glomeruli were in the I-II category (96%) and only 1.7–3% in the III-IV category. However, there was a significant increase in structural change in the T and OVX+T groups with 9.7–15.6% in category III-IV and 89–83%, respectively, in category I-II.

## 4. Discussion

### 4.1. Blood Pressure

In the present study, we demonstrated that E removal (ovarectomy) combined with T (OVX+T) treatment significantly elevated BP in female SHRs compared to controls, OVX, or T groups all on a high sodium diet (3%). This represents a male pattern of BP rise with time. For instance, in a previous study we showed that BP rose in SHR males between 5 and 15 weeks from 138 to 190 mmHg or about 5 mmHg/week [23]. The SHR females in the present study in the OVX+T group rose 145–190 mmHg in 9 weeks or about 5 mmHg/week. The BP pattern of the OVX+T group rose weekly until the last 3 weeks and then reached a plateau. The control and OVX groups reached a peak BP about week 11 and then leveled off, whereas the T group had a decrease in BP after week 11 and a decline from 170 to 150 mmHg at week 14. The drop in BP in the T group from week 11 to 12 and 11 to 13 was not significant, but the difference between week 11 and week 14 was significant (*P* < .05). The explanation for this decrease is not known, but the T implant was not the problem. Every two weeks, the implants were replaced and there was always T remaining in the implant. Also there were no changes in environmental conditions that could explain the fall in the BP. Possibly, there was a compensation by E to reduce the BP by vasodilation since this group still had ovarian function. Using a normal sodium diet, Chen and Meng [24] found similar results regarding the influence of T on the development of hypertension in both male and female SHR during 4–20 weeks of age. In order to explain potential mechanisms for the BP effect, the influence of the high sodium diet should be examined. Caplea et al. [22] showed that a high sodium diet increased plasma NE by 71% compared to animals on a control sodium diet and treatment with clonidine normalized the plasma NE rise. Since all of the groups were on the same high sodium diet this variable cannot explain the group differences. The plasma level of NE for all groups is high compared to NE levels collected from conscious SHR rats due to several factors: the movement of the cages to a new room could activate the SNS and release more NE; application of an anesthetic can also activate the SNS and the animals were on a high sodium diet which elevates plasma NE [22]. We get plasma NE values of about 1500 pg/mL from male SHR in a colony environment using this technique compared to 2500–3000 pg/mL in these experiments with females [21]. Also we have shown that a high sodium diet alone does not increase BP in SHR, if the animals are not socially stressed by interacting in a colony environment (open field caging with males and females interacting) [22, 25]. A colony environment combined with a high sodium diet did significantly raise telemetered BP in both normotensive WKY and hybrid SHR males but not without the colony stimulation [22]. This brings us to the involvement of T in the BP effect. In the above study by Caplea et al. [22] when the colony males (socially interacting in an open field environment) were given an androgen receptor antagonist (flutamide) the nocturnal rise in BP was eliminated in spite of the high sodium diet suggesting that T when bound to the androgen receptor produced the elevated BP. We know that castration lowers BP in males and T replacement increases BP [14–16]. Not only T, but also the presence of androgen receptors are necessary for the development of hypertension. Hybrid SHR males (the male offspring of King Holtzman females carrying the testicular feminized mutation bred with an SHR male) without functional androgen receptors had significantly reduced BP compared to those with functional androgen receptors [17].

### 4.2. Testosterone-Norepinephrine Interaction

So if plasma NE is elevated by T and T can influence BP, is there evidence of a T-NE interaction? In SHR males, Jones et al. showed that T enhanced the renal fractional release of NE (>100% compared to castrates) from the kidney [26]. Kumai et al. established that androgens administered to castrated SHR males raised tyrosine hydroxylase in the adrenal medulla [27] and in the aorta and mesenteric arteries [28] leading to increased NE and elevated BP. T can elevate BP by increasing the responsiveness of resistance vessels to sympathetic stimulation through several mechanisms. For instance, T increases *α*-adrenergic receptors in smooth muscle cells [29], and T increases the release of the vasoconstrictor norepinephrine (NE) [30]. Our laboratory has verified this result and found that T increased renal NE content and fractional release of NE in response to renal nerve stimulation [26]. This may be an important mechanism which enhances the reabsorption of sodium in the kidney [31]. T has been implicated in reduced renal sodium excretion and may involve blunting of the pressure-natriuresis relationship [31]. Other investigators have demonstrated that T causes a delayed inactivation of NE via inhibition of the NE up-take mechanism [32], and this action would increase the availability of NE to bind to alpha receptors on blood vessels and promote vasoconstriction.

### 4.3. Estrogen

E also has a role in BP regulation in the female. E relaxes blood vessels by increasing the synthesis [33] and release [34] of nitric oxide, a potent endothelial vasodilator [35]. Moreover, E can relax coronary arteries by stimulating the gating of the large-conductance Ca^++^ and voltage-activated K^+^ channels in smooth muscle, which retards the contractility of this tissue [36]. Our data suggest that E had some protective effect on BP since T did raise BP as much if the ovaries were present compared to when they were absent. E may also be protective against hypertension by amplifying the vasodilator contributions of angiotensin (1–7), while reducing the formation and vasoconstrictor actions of angiotensin II [37]. Some of the protective effects of E may be negated by T in that T administered to macrophages decreased nitrite release by decreasing inducible nitric oxide synthase which could increase platelet aggregation and thrombosis risk [38].

Based on the results of this study, we could not exclude the effects of ovarian hormones other than E, such as progesterone, on BP in the two groups with ovaries. However, the day that BP measurements were taken was random with regard to the ovarian cycle which should minimize interference on BP due to progesterone. It is important to note that with the OVX procedure, not only is E lost, but also other ovarian hormones are removed which could have an impact on BP and renal function.

### 4.4. Sodium Reabsorption

With regards to sodium reabsorption, the data show that both E and T increased renal sodium reabsorption. The OVX group does not have E but it does have some adrenal gland produced T; the T group has both E and T present, and the OVX+T group has mostly T present. In all these groups, sodium reabsorption increased. Is there data suggesting that these steroids can influence renal sodium processing? Castration increases sodium excretion and T replacement restores sodium excretion to control levels in both WKY and SHR/y males [39]. Also defective androgen receptors in the kidney and antiandrogens like flutamide increased Na excretion [39, 40]. In six-month-old female Sprague-Dawley rats, OVX resulted in decreased sodium excretion (>50%) compared to controls suggesting an E effect [41]. Potential mechanisms for the E effects on sodium excretion may be through both AT_1_ and AT_2_ receptors and the renal epithelial sodium channel (ENaC). In WKY females, AT_1_ and AT_2_ receptor expression was E dependent [42]. In Wistar rats, ENaC subunit expression was higher in females than males, and the differences were abolished by OVX and restored by E [43]. Since ENaC is the primary route for sodium absorption in the mammalian distal tubule, it is probable that it plays an important role in sodium handling and is regulated by E.

### 4.5. Renal Histology

With regard to renal injury, one potential mechanism to explain the T effect on BP and the kidney is the interaction between T and the sympathetic nervous system (SNS). We have reported that neonatal sympathectomy reduced glomerulosclerosis scores in our consomic Y chromosome strain that has a WKY genetic background with an SHR Y chromosome [44]. Also we have shown that females with an SHR autosomal background have a higher renal NE turnover rate than males of the same strain [45]. This suggests that the females may have more renal sympathetic activity but be protected from renal injury in the presence of E. However, when E is removed or T is added in the presence of a high sodium diet, there is further increased SNS activity and acceleration of renal injury. In another model using Sprague Dawley rats, elevated SNS activity (induced by phenylephrine infusion) produced salt-dependent hypertension (measured by telemetry) after cessation of the infusion and renal injury [46]. The injury was not primarily glomerular but tubular-interstitial. NE can cause glomerular damage by producing renal ischemia and glomerular collapse and damage can be caused by very high glomerular hydrostatic pressure generated by elevated NE [47]. 

The combination of T and NE impacting the kidney may be more important for initiating renal injury than elevated BP. A large body of evidence supports the concept that renal dysfunction occurs first, followed by the development of hypertension [48]. Reckelhoff and Granger have postulated that a combination of increased T and angiotensin II increased renal Na reabsorption and elevated glomerular capillary pressure produce glomerular injury [40]. High BP does not appear to play the only role in the development of renal pathology since ACE inhibition controlled BP effectively in SHR but only postponed the onset of kidney disease [49]. Indeed, in male SHR when we lowered the BP with hydralazine but did not alter T, we still observed renal pathology [16]. In SHR male controls, the highest glomerular damage resulted after 10 weeks when BP approached 220 mmHg. Removal of T significantly reduced BP and renal damage. In addition, removal of T combined with hydralazine further reduced glomerular damage but not BP. When T was added after castration, BP and glomerular damage increased [16].

Also, normotensive virgin Munich-Wistar females and males castrated at 10 weeks of age were protected from renal injury during aging (18–20 months) [50]. Females were more protected than males which may have been due to a metalloproteinase protection in females [50]. Similarly, in normotensive ageing Wistar and Sprague-Dawley rats, castration of the male prevented the age-related glomerulosclerosis which supports a mechanism that does not necessarily require high blood pressure in order for glomerulosclerosis to occur but does require T [51]. 

 In rats, transgenic for both the human renin and angiotensinogen genes, hypertension and kidney damage developed which was largely independent of BP elevation but was dependent on an androgen component [51]. For instance, androgen receptor blockade (flutamide) significantly attenuated the development of hypertension in females and reduced plasma renin activity and rat renin mRNA in the kidney [52]. Urinary albumin excretion was blunted, collagen III mRNA was significantly decreased, and no histological characteristics of end-organ damage were observed in the kidney after treatment [52]. These results demonstrated that when the androgen receptor is blocked, there is protection against hypertension and end-organ damage not only in male but also in female transgenic rats (TGRmREN27) [52].

 In summary, exogenous T and the absence of ovarian estrogen increased BP in SHR females producing a male pattern of BP rise which resulted in reduced sodium excretion, elevated plasma NE and renal pathology. The loss of E alone did not cause a rise in BP, plasma NE, or renal pathology. However, the combination of the loss of E and the addition of T contributed to the elevated BP raised plasma NE and renal glomerular changes.

## Figures and Tables

**Table 1:** Glomerular scores by treatment and severity.

Group	0	I-II	III-IV
Control (*n* = 8)	1.9 ± 0.6	96.4 ± 0.8	1.7 ± 0.2
OVX (*n* = 6)	0.6 ± 0.08	96.4 ± 0.9	3.0 ± 1.0
Testosterone (*n* = 6)	1.0 ± 0.4	89.3* ± 2.1	9.7* ± 2.4
OVX +T (*n* = 7)	1.4 ± 1.0	83.0** ± 2.8	15.6** ± 3.4

(means, ± S.E.M., *P < .05, **P < .01 compared to controls, OVX: ovarectomy, OVX+T: ovarectomy and testosterone treatment, 0: normal glomerulus, I-II: moderate glomerulosclerosis, III-IV: severe glomerulosclerosis).
